# Birch pollen allergen-induced dsDNA release activates cGAS-STING signaling and type 2 immune response in mice

**DOI:** 10.1016/j.isci.2025.112324

**Published:** 2025-03-31

**Authors:** Pauline Chenuet, Manon Mellier, Yasmine Messaoud-Nacer, Elodie Culerier, Quentin Marquant, Louis Fauconnier, Nathalie Rouxel, Aurélie Ledru, Stéphanie Rose, Bernhard Ryffel, Lionel Apetoh, Valérie F.J. Quesniaux, Dieudonnée Togbe

**Affiliations:** 1 Artimmune SAS, 13 Avenue Buffon, 45100 Orléans, France; 2 Laboratory of Immuno-Neuro Modulation (INEM), UMR 7355 CNRS and University of Orleans, 3B rue de la Ferollerie, 45071 Orleans-Cedex, France; 3 University of Orleans, 45000 Orleans, France; 4 Brown Center for Immunotherapy, Indiana University Melvin and Bren Simon Comprehensive Cancer Center, Indiana University School of Medicine, Indianapolis, IN 46202, USA

**Keywords:** Immune response, Immunology, Molecular biology

## Abstract

Detecting cytoplasmic or extracellular DNA from host or pathogen origin by DNA sensor cyclic GMP-AMP synthase (cGAS) and stimulator of interferon genes (STING) triggers immune responses with secretion of type I interferons and inflammatory cytokines. However, STING agonists function as type-2 adjuvant promoting allergic asthma. Here, we asked how cGAS/STING signaling pathway influences allergen-induced type-2 immune responses in models of allergic airway diseases induced by birch pollen extract, house dust mite, or ovalbumin plus Alum. We report increased extracellular dsDNA in the airways, together with cGAS and STING gene expression, following allergen challenge in these models, correlating dsDNA and type-2 cytokine IL-4, IL-5, and IL-13 release. Allergen-induced type-2 immune responses were reduced in cGAS- or STING-deficient mice. Further, blocking cGAS function with the specific inhibitor RU.521 protected mice from birch pollen allergen-induced airway inflammation and type-2 immune responses. Thus, DNA sensing by cGAS contributes to type-2 immune responses and may represent a therapeutic target for allergic lung inflammation.

## Highlights

• Allergens trigger airway dsDNA release and DNA sensor cGAS-STING expression
• Allergen-induced dsDNA release correlates with type 2 cytokines in the airways
• Reduced allergen-type 2 immune response in cGAS- or STING-deficient mice
• cGAS inhibitor protects from allergen airway inflammation and type 2 response

## Graphical abstract

**Figure:**
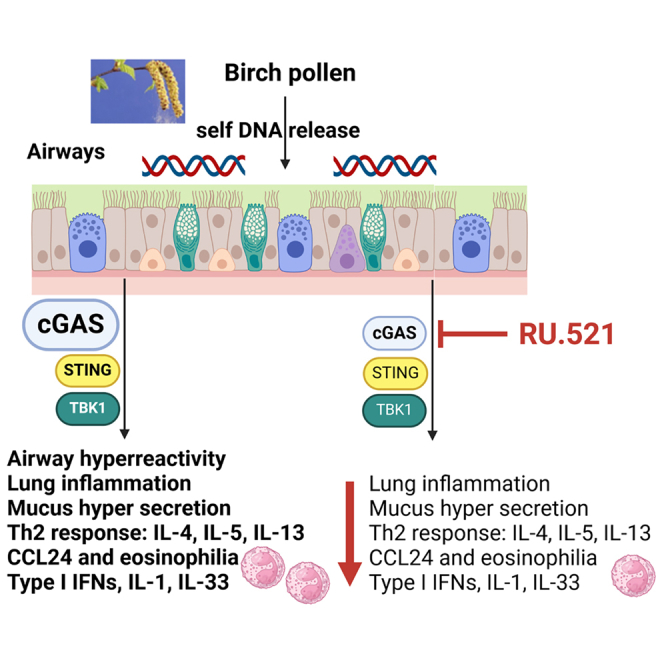

## Introduction

In eukaryotes, self-DNA localization is restricted to the cell nucleus and mitochondria, thereby sequestering self-DNA from DNA sensing mechanisms that may activate pro-inflammatory pathways. Self-DNA released from injured cells or tissues constitutes a potent danger signal. DNases eliminate aberrant self-DNA found in apoptotic bodies, extracellular space, cytosol, and endosomes.^1^^,^^2^ Pollens contain proteases that induce cell and tissue damage, leading to ectopic DNA release. Extracellular self-DNA, with increased mitochondrial DNA (mtDNA) to nuclear DNA (nDNA) ratio, is a biomarker of chronic lung inflammation in allergic asthma.^3^ Increased double-stranded DNA (dsDNA) in the bronchoalveolar lavage from asthmatic patients with rhinovirus infection was reported to promote asthma exacerbation.^4^

In experimental asthma animal models, adjuvants such as aluminum salts alum are rapidly coated by host DNA released from dying cells, acting as a damage-associated molecular pattern (DAMP).^5^^,^^6^ Mice deficient for stimulator of interferon genes (STING) showed a reduced antigen-specific IgE response following immunization with ovalbumin plus alum,^6^ suggesting that self-DNA and STING activation contribute to alum adjuvant activity.^5^^,^^6^ Further, the vaccine adjuvant chitosan induces mitochondrial stress with mtDNA release into the cytosol in mouse dendritic cells, engaging cyclic GMP-AMP synthase (cGAS) and STING pathway to induce type I interferons (IFNs) and protective immunity.^7^

Self-DNA might act at several levels, inducing primary B cell responses and IgG1 production, through IRF3-independent mechanisms, while stimulating T helper type 2 responses through IRF3-dependent mechanisms.^5^ However, the role of the DNA sensor cGAS/STING *per se* in allergic asthma is not fully characterized, as well as the relative role of self-dsDNA-induced type I IFNs on the Th2 responses.

STING ligand 2′3′ cyclic GMP-AMP (cGAMP) acts as a type 2 adjuvant promoting allergic asthma in a way dependent on STING/TBK1/IRF3/7 signaling pathway and IL-33/ST2.^8^ Further, STING plays an important role in the maturation and class switching of IgE-producing B cells in allergic inflammation via follicular helper T (Tfh) cells.^9^

The implication of epithelial cGAS in murine models of allergic asthma was reported,^10^ as cytosolic dsDNA accumulated in the airway epithelium of ovalbumin- or house dust mite-challenged mice, and deletion of cGAS in airway epithelial cells attenuated allergic airway inflammation.^10^ However, there was no direct demonstration of extracellular DNA release in the airways, nor comparison of cGAS vs. STING implication in airway allergic responses.

Here, we addressed self-DNA release and the contribution of cGAS/STING signaling pathway on allergen-induced type 2 immune responses in models of allergic airway diseases. We induced allergic airway disease by birch pollen extract or house dust mite without adjuvant, or by ovalbumin plus alum adjuvant, using cGAS- and STING-deficient mice, and tested the efficacy of a cGAS inhibitor. We report dsDNA release in the airways following allergen challenge, irrespective of adjuvant usage, correlating with type 2 cytokine release. The type 2 response and airway hyperresponsiveness (AHR) were affected in the absence of cGAS and to a lesser extent of STING. Further, pharmacological blocking of cGAS protected from birch pollen-induced type 2 immune responses, validating the contribution of cGAS to allergen-induced type 2 immune responses that may represent a potential therapeutic target for allergic asthma.

## Results

### Allergen exposure induces dsDNA release in the airways and DNA sensor cGAS/STING gene expression in the lung

**Figure 1:**
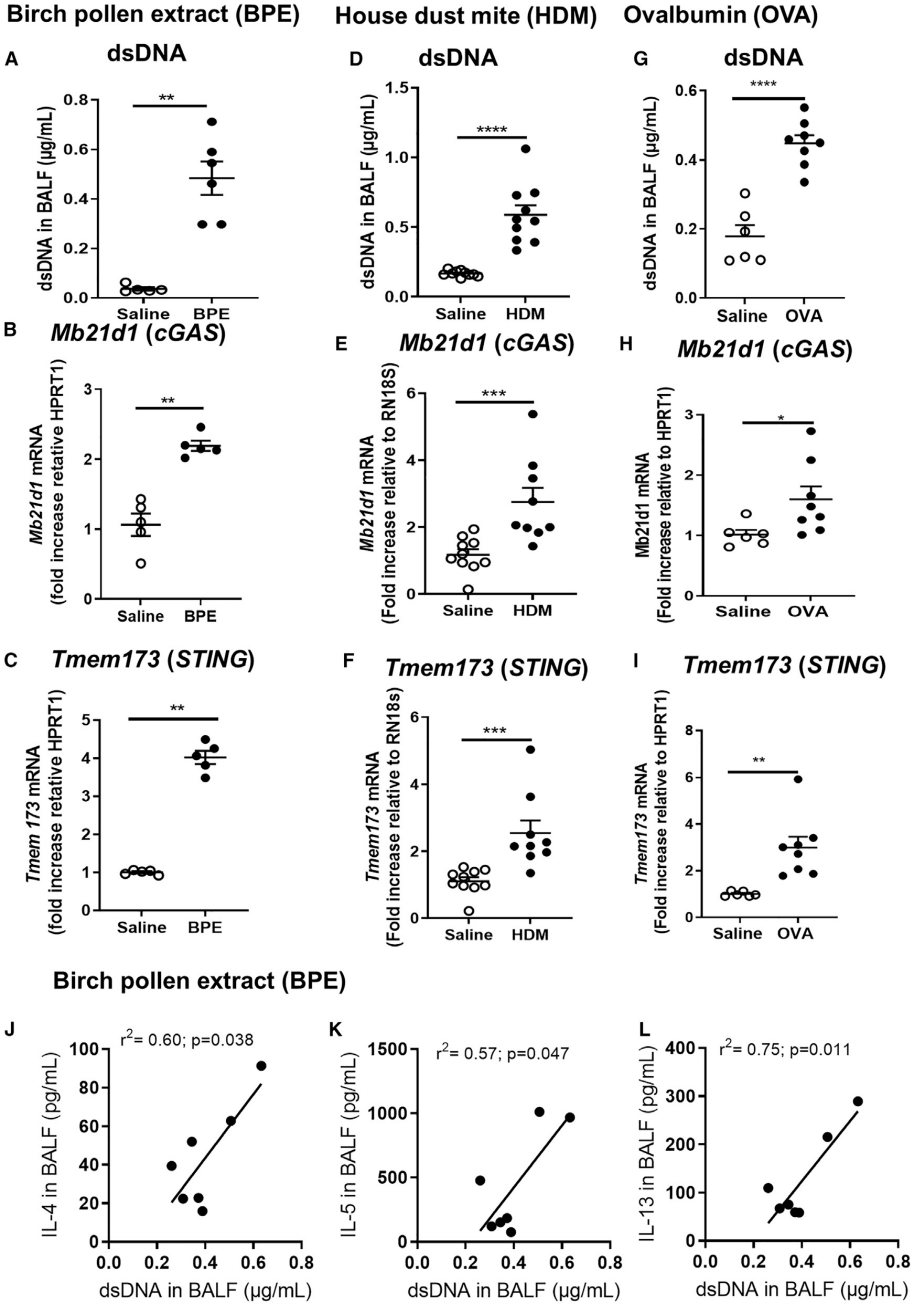
Allergen exposures trigger dsDNA release and cGAS-STING pathway mobilization (A–I) Wild-type (WT) mice were exposed to birch pollen extract (BPE; A–C), house dust mite (HDM; D–F), and ovalbumin with alum (OVA; G–I) and DNA sensing pathway characterized. Mice were immunized with BPE (10 μg/mouse i.p. on day 0 and 14) and challenged daily from day 21–24 (10 μg/mouse i.n.). Alternatively, mice were sensitized with HDM (25 μg i.n.) on day 0 and 7, followed by three HDM challenges (10 μg/mouse i.n.) on day 14–16. Immunization with OVA (20 μg/mouse emulsified in 2 mg Alum gel i.p.) on day 0, 7, and 14 was followed by challenges on day 21–24 (OVA 10 μg/mouse i.n.). Control mice received saline solution. (A–I), Extracellular dsDNA concentration in the acellular fractions of bronchoalveolar lavage fluid (BALF), *Tmem173* and *Mb21d1* transcripts in the lungs normalized to *RN18s* or *Hprt1* expression. (J–L) Correlation of dsDNA and IL-4, IL-5, and IL-13, respectively, in BALF of BPE exposed mice. Data are representative of two independent experiments. Graph data in (A–I) are presented as mean ± SEM with *n* = 5–10 mice/group in the saline vehicle control and 5–10 mice/group for the allergen-exposed group. Each point represents an individual mouse. Statistics were performed by non-parametric Mann-Whitney test (A–I), and by Spearman for correlation analysis (J–L). *p* value < 0.05 (∗); *p* value < 0.01 (∗∗), *p* value < 0.001 (∗∗∗), *p* value < 0.0001 (∗∗∗∗).

We hypothesized that air-born allergens may cause airway cell stress and death with DNA release and mobilize DNA sensing pathways. To study whether allergen exposure leads to extracellular DNA release and gene expression of nucleic acid sensors, we tested the effect of three different sets of allergens: birch pollen extract (BPE; Figures 1A–1C), house dust mite (HDM; Figures 1D–1F) without adjuvant, and ovalbumin (OVA) plus alum adjuvant (Figures 1G–1I). All three allergens BPE, HDM, and OVA induced significant extracellular DNA release in the bronchoalveolar space of C57BL/6J mice (Figures 1A, 1D, and 1G). Concomitantly, there was an increased pulmonary expression of DNA sensor cGAS gene *Mb21d1* (Figures 1B, 1E, and 1H) and STING gene *Transmembrane Protein 173*, *Tmem173* (Figures 1C, 1F, and 1I). Allergen exposure leads to type 2 immune response, and we next investigated how extracellular DNA release might influence type 2 cytokine release in the airways of mice exposed to birch pollen. We found a strong correlation between dsDNA and IL-4, IL-5, or IL-13 concentrations in the airways of BPE challenged mice (Figures 1J–1L).

Thus, exposure to environmental allergens induced extracellular DNA release in the airways and upregulation of cGAS/STING.

### cGAS/STING signaling is required for birch pollen induced allergic lung Th2 responses

**Figure 2:**
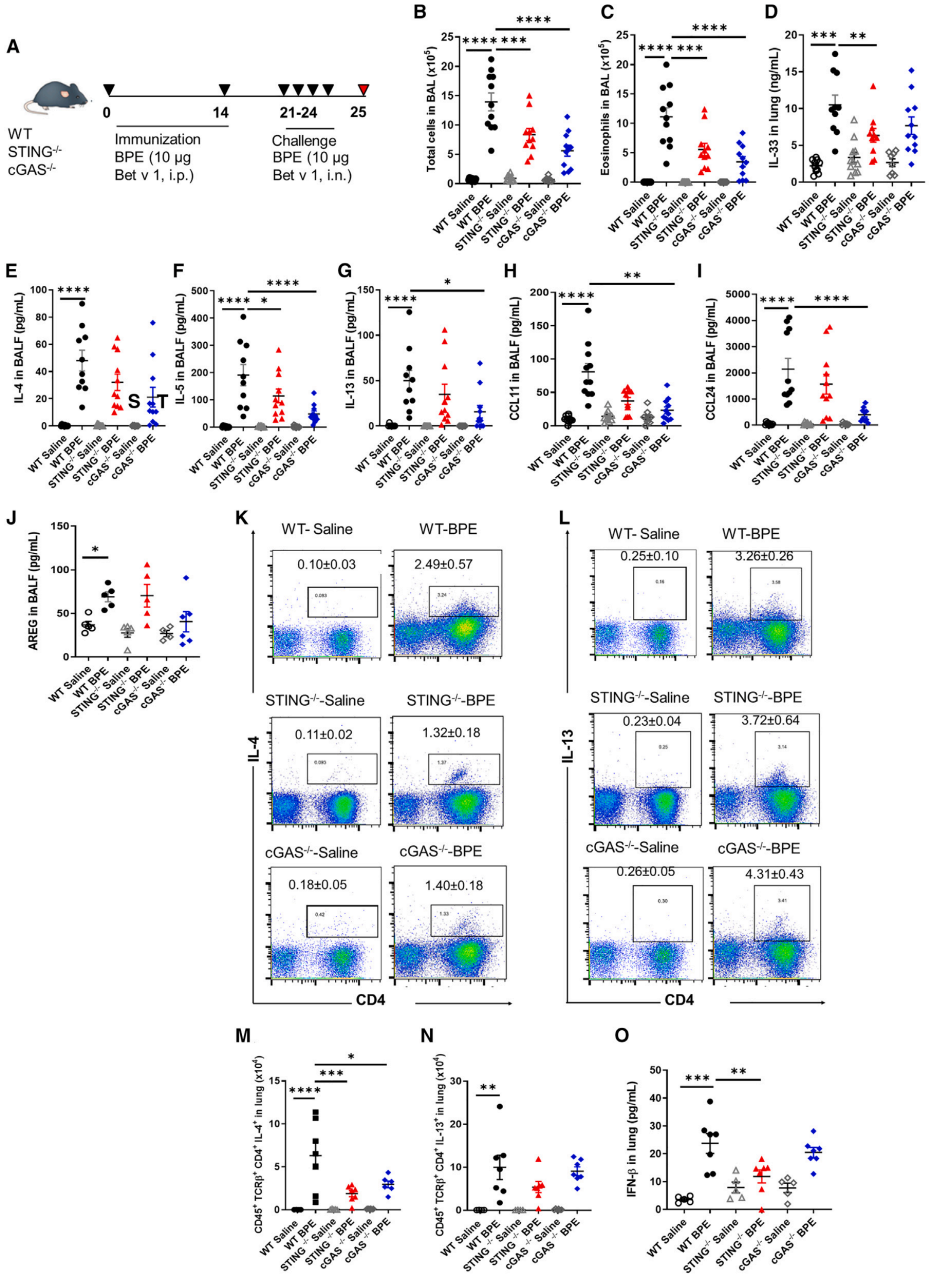
Birch pollen induces a cGAS/STING-dependent lung inflammation (A) WT, cGAS^−/−^, and STING^−/−^ mice were sensitized twice with i.p. injections of birch pollen extract (BPE, equivalent to 10 μg Bet v 1) on days 0 and 14, followed by four intranasal BPE challenges (10 μg Bet v 1) daily on day 21–24, and analyzed on day 25 (A). Control mice were immunized and challenged with saline alone. (B and C) Total cell and eosinophil counts in BALF determined by May-Grünwald Giemsa staining of BAL cell cytospins. (D–G) IL-33, IL-4, IL-5, and IL-13 in BALF. (H–J) CCL11, CCL24, AREG concentrations in BALF. (K–N) Representative FACS dot plots with frequency and absolute number of CD4^+^IL-4^+^ and CD4^+^IL-13^+^ cells in lung. (O) Type I IFNβ concentration in BALF. Data are pooled from two independent experiments and are presented as mean ± SEM with *n* = 5–10 mice/group in the saline vehicle control and 7–11 mice/group for the BPE-exposed group. Each point represents an individual mouse. Statistics were performed by using one-way ANOVA test followed by Sidak multiple comparison test (A–C, F, I, J, and M–O) or non-parametric Kuskal-Wallis test followed by Dunn multiple comparisons test (E, G, and H). *p* value < 0.05 (∗); *p* value < 0.01 (∗∗), *p* value < 0.001 (∗∗∗), *p* value < 0.0001 (∗∗∗∗). See also Figure S1.

We next questioned the role of cGAS/STING pathway in allergic airway inflammation. To study how the absence of functional cGAS/STING pathway may affect the pathogenesis of allergic airway inflammation, we analyzed the response of mice deficient for cGAS (cGAS^−/−^) or STING (STING^−/−^), vs. wild-type (WT) mice to a well-established model of allergic lung inflammation induced by birch pollen administration. To this end, mice sensitized with BPE on day 0 and 14 (10 μg, i.p.) were challenged with BPE intranasally on 4 consecutive days (10 μg, i.n. on day 21–24). Mice were analyzed on day 25, 24 h after the last BPE instillation (Figure 2A). WT mice developed all the cardinal features of asthma, including immune cell infiltration in the bronchoalveolar space, with increased total cells and eosinophils (Figures 2B and 2C), and increased secretion of the epithelial-derived alarmin IL-33 (Figure 2D), of the Th2 cytokines IL-4, IL-5, and IL-13 (Figures 2E–2G) and of the eosinophil-attracting chemokines CCL11 and CCL24 (Figures 2H and 2I).

In contrast, cGAS^−/−^ mice, and less prominently STING^−/−^ mice exhibited reduced inflammatory phenotype characterized by lower total cell infiltration, eosinophilia, and Th2 cytokines or chemokines release in the airways (Figures 2B–2I). Amphiregulin (AREG), a critical component of type 2 mediated tissue repair,^11^ was increased after BPE challenge, and less so in cGAS^−/−^ mice, although this did not reach statistical significance, while it was not affected in STING^−/−^ mice (Figure 2J).

We next questioned how BPE-induced Th2 response may be affected in the absence of cGAS or STING. Lung cells of mice primed and challenged with BPE as aforementioned were gated on CD45^+^TCRβ^+^ singlet cells (Figure S1) and IL-4 and IL-13 secreting CD4^+^ T cells analyzed. The strong increase of IL-4^+^ CD4^+^ T cells seen after BPE challenge in WT mice was prevented in both STING^−/−^ and cGAS^−/−^ mice, while IL-13^+^ secreting CD4^+^ T cells were not affected (Figures 2K–2N).

As extracellular DNA was present in the airways and DNA sensor cGAS/STING pathway upregulated after allergen challenge, we next asked whether type I IFNβ was induced. Indeed, IFNβ was increased in the lung tissue of WT mice challenged with BPE (Figure 2O) and less so in STING^−/−^ mice (Figure 2O).

Thus, deletion of cGAS/STING pathway reduced BPE-induced eosinophil recruitment, Th2 responses, and type I IFN, which appears to be the result of the coordinated actions of both local innate and Th2 adaptive immune responses.

### DNA sensor cGAS drives airway hyperresponsiveness and lung histopathology

**Figure 3:**
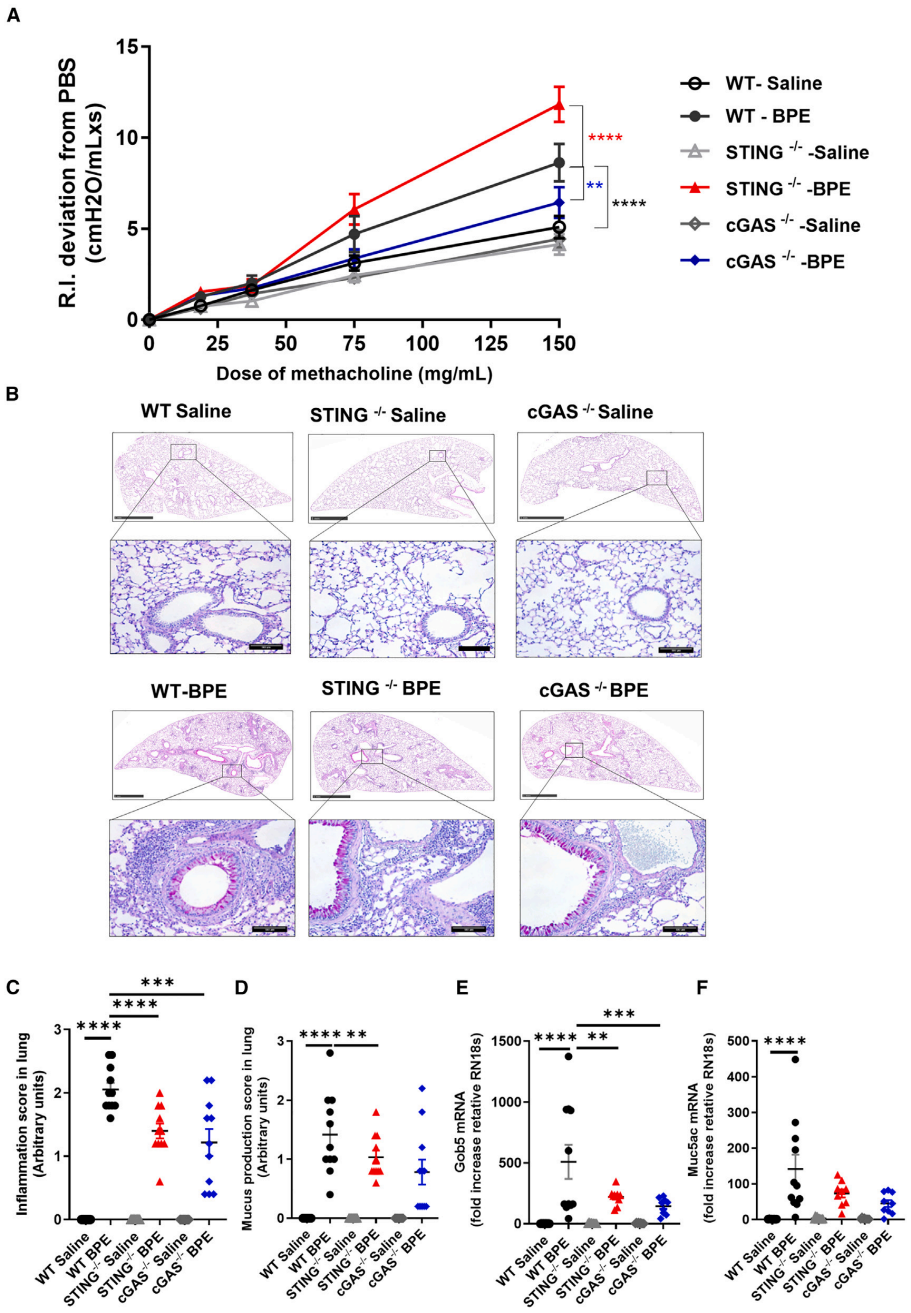
cGAS/STING-dependence of birch pollen-induced airway resistance WT, cGAS^−/−^, and STING^−/−^ mice were treated as in Figure 2 and analyzed 24 h after the last intranasal birch pollen extract (BPE) allergen challenge or saline as vehicle control. (A) Airway resistance was measured with increasing dose of methacholine using a FinePointe system (Buxco). (B–D) Lung tissue histology with Periodic acid Schiff (PAS) staining of goblet cells for mucus production (B) (scale bar 1 mm for low magnification and 100 μm for ×20 higher magnification), with pathology scoring of the peribronchial infiltration of inflammatory cells (C) and mucus production (D). (E and F) *Gob5* and *Muc5ac* gene expression were measured in lung tissue. Data are presented as mean values ± SEM of *n* = 9–10 animals in the saline group and 9–11 animals for BPE group. Statistics were performed by two-way ANOVA test followed by Tukey multiple comparisons test (A), one-way ANOVA test followed by Sidak multiple comparison test (C–E) or non-parametric Kuskal-Wallis test followed by Dunn multiple comparisons test (F). ns: not significant. *p* value < 0.05 (∗); *p* value < 0.01 (∗∗), *p* value < 0.001 (∗∗∗), *p* value < 0.0001 (∗∗∗∗).

In allergic asthma, the degree of inflammation is thought to correlate with airflow obstruction and AHR development due to the ensuing activation of smooth muscle cells. Mice sensitized with BPE on day 0 and 14 and challenged intranasally with BPE on day 21–24 as aforementioned were analyzed 24 h after the last BPE instillation. Lung resistance increased in BPE challenged WT mice, as seen under increasing methacholine exposure (Figure 3A). This BPE-induced increase of lung resistance was attenuated in cGAS^−/−^ mice, as compared to WT mice (Figure 3A), while deletion of STING resulted in a worsening of AHR resistance (Figure 3A).

The histopathological examination of lung sections revealed an infiltration of inflammatory cells around the airways and blood vessels which expanded into the parenchyma in BPE exposed WT mice, with the presence of dense cellular aggregates around the airways (Figures 3B and 3C). The cellular infiltrate consisted mainly of eosinophils (Figure 3B). Histopathologic analysis of periodic acid Schiff (PAS)-stained lung sections, together with RT-qPCR analysis of *Muc5AC* and *Gob5* mRNA revealed an increased mucus hypersecretion in the airways of BPE exposed WT mice (Figure 3B and 3D–3F). There was a reduced inflammatory cell infiltration into the parenchyma in BPE exposed cGAS^−/−^ and STING^−/−^ mice, along with a reduction in mucus production and lower expression of *Gob5* and *Muc5AC* (Figures 3B–3F).

Thus, BPE challenge induced AHR, lung inflammation with mucus hypersecretion contributing to airway obstruction which was reduced in the absence of cGAS/STING.

### cGAS-specific inhibitor limits birch pollen induced allergic lung inflammation and type 2 immune responses

**Figure 4:**
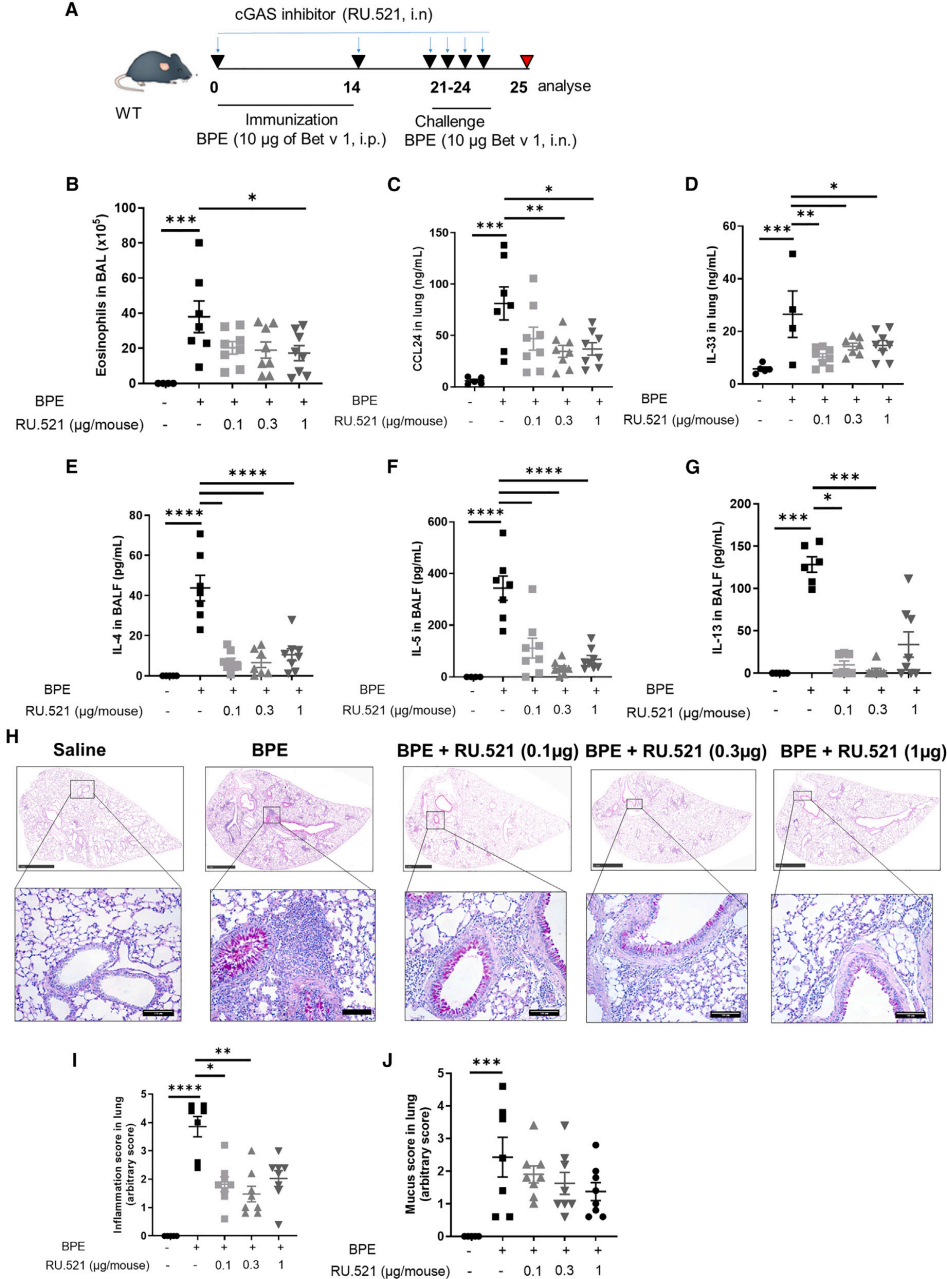
Selective inhibition of cGAS mediated signaling by RU.521 reduces type 2 immune response and lung inflammation WT mice were sensitized twice with i.p. injections of birch pollen extract (BPE, using doses equivalent to 10 μg Bet v 1) on day 0 and 14, followed by four daily challenges on day 21–24 of intranasal BPE (equivalent to 10 μg Bet v 1) under Isoflurane anesthesia (3%). (A) cGAS inhibitor (RU.521; at the doses of 5, 15, and 50 μg/kg Invitrogen) was administered to mice by i.n. route during sensitization and challenge. Twenty-four hours after the last BPE challenge, differential cell counts were determined in BAL. (B) Eosinophil counts in BAL. (C–G) Concentrations of CCL24 and IL-33 in lung homogenates, and IL-4, IL-5, and IL-13 in BALF. (H–J) PAS stained lung sections of goblet cells for mucus production (H) (scale bar 1 mm for low magnification and 100 μm for ×20 higher magnification), with pathology scoring of the peribronchial infiltration of inflammatory cells (I) and mucus production (J). Data are presented as mean values ±SEM of *n* = 5 animals in the saline group and 7–8 animals for BPE group. Statistics were performed by one-way ANOVA test followed by Sidak multiple comparison test (B–D and J) or non-parametric Kuskal-Wallis test followed by Dunn multiple comparisons test (G and I). *p* value < 0.05 (∗); *p* value < 0.01 (∗∗), *p* value < 0.001 (∗∗∗), *p* value < 0.0001 (∗∗∗∗). See also Figure S2.

To further evaluate the contribution of cGAS to allergic lung inflammation, we next used a specific cGAS inhibitor *in vivo* in the model of birch pollen induced allergic lung inflammation. cGAS inhibitor RU.521^12^ was administered at 0.1, 0.3, and 1 μg/mouse by intranasal route during sensitization and challenge of WT mice on day 0, 14, and 21–24 (Figure 4A). The increase in eosinophils induced after BPE challenge in the airways was reduced by RU.521 treatment (Figure 4B), as was the release of eosinophil-attracting CCL24 and IL-33 in the bronchoalveolar lavage fluid (BALF) (Figures 4C and 4D). The induction of Th2 cytokines IL-4, IL-5, and IL-13 in the BALF was also prevented by RU.521 (Figures 4E–4G). Histopathologic analysis of PAS-stained lung sections showed reduced expansion of inflammatory cell infiltration into the parenchyma following BPE challenge with RU.521 treatments, with a partial, non-significantly reduced mucus overproduction (Figures 4H–4J).

Therefore, pharmacological blocking of cGAS prevented BPE-induced eosinophil recruitment and Th2 responses, leading to reduced lung inflammation.

## Discussion

There are several indications of self-DNA release during allergic asthma. This includes increased extracellular, self mtDNA/nDNA ratio which serves as a biomarker of chronic lung inflammation in allergic asthma,^3^ or increased dsDNA in the BALF of patients with asthma together with rhinovirus infection.^4^ Extracellular dsDNA in the BALF may reflect upstream cell stress and death, with leakage of mtDNA in the cytoplasm, formation of cytoplasmic micronuclei that activate the cGAS-STING pathway^13^ and transport of mtDNA and nDNA outside of the cell by different mechanisms. Extracellular DNA may re-enter the cytoplasm via endocytosis after binding to HMGB1, as reported in classical dendritic cells (cDC),^14^ or via extracellular vesicles (EVs) loaded with DNA from diseased cells being internalized and activating STING-dependent type I IFN response.^15^ The implication of airway epithelial cGAS DNA sensor was reported in murine models of allergic asthma.^10^ Indeed, there was an increased accumulation of cytosolic dsDNA in the airway epithelium of OVA or HDM-challenged mice, and the deletion of cGAS in the airway epithelial cells attenuated OVA or HDM induced allergic airway inflammation.^10^ However, there was no direct comparison of cGAS vs. STING implication in airway allergic responses.

Here, we report that cGAS/STING signaling pathway influences allergen-induced type 2 immune responses in models of allergic airway diseases induced by birch pollen extract, HDM, or OVA plus alum. Indeed, exposure to these environmental allergens induced both extracellular DNA release in the airways and cGAS/STING mobilization through increased cGAS and STING pulmonary gene expression. After birch pollen extract challenge, extracellular dsDNA correlated with type 2 cytokines release in the airways. Allergen-induced eosinophil recruitment and type 2 immune responses including IL-4^+^CD4^+^Th2 cells were significantly decreased in mice deleted in cGAS or STING. The differences observed in STING- and cGAS-deficient mice may reflect the specific and non-overlapping roles of STING and cGAS in immune signaling and airway sensitivity. In cGAS^−/−^ mice, inflammation and airway irritant responses were reduced, thus limiting STING activation and subsequent immune cell recruitment. In STING^−/−^ mice, the reduced inflammation was accompanied by increased bronchial irritant response that may be linked to compensatory pathways or increased sensory neuron reactivity. Indeed, airway hyper-responsiveness depends upon inflammatory mediators including histamine, prostaglandin D2, leukotrienes, or cytokines, such as IL-4, IL-13, Thymic Stromal Lymphopoietin (TSLP), and IL-33 but may also be influenced by airway remodeling or neuronal dysfunction. Recent transcriptional analysis of sensitized sensory neurons from CFA-treated mice showed increased STING expression in Nav1.8^+^ and TRPV1^+^ neurons.^16^ Increasing evidence demonstrate the essential function of transient receptor potential vanilloid family (TRPV1, 2, and 4) in the pathogenesis of asthma. TRPV1 is associated with the major features of asthma, such as inflammation, AHR, and remodeling.^17^ Thus, the specific enhancement of bronchial irritant response in STING^−/−^ mice might be linked to STING regulation in sensory neurons. Further investigations may provide insight into the mechanism of STING expression on TRPV1^+^ cells and the regulation of airway hyperesponsiveness.

STING ligand cGAMP and STING itself have been implicated as Type 2 response inducers in allergic asthma models. Indeed, cGAMP functions as a type 2 adjuvant promoting allergic asthma in a way dependent on STING/TBK1/IRF3/7 signaling pathway and IL-33/ST2.^8^ Further, STING was reported to play an important role in the maturation and class switching of IgE-producing B cells in allergic infammation via Tfh cells.^9^

The field of DNA sensing has been expanding over the past decade, and emerging dimensions of cGAS/STING involvement in cellular responses are still becoming more complex.^18^ In particular, cGAS likely exerts a structural function by forming cGAS-DNA complex, first as 2:2 cGAS-DNA assembly, two cGAS molecules embracing two strands of DNA via A- and B-site DNA-binding surfaces of the NTase,^19^ then by further assembly via cross-bridged arrangements on long DNA stretches.^18^ This, plus further multivalent interactions between cGAS and DNA, via the unstructured, positively charged N terminus result in the formation of biomolecular condensates through liquid-liquid phase separation (LLPS). It is likely that the total absence of cGAS protein in cGAS^−/−^ mice will not allow this high order DNA complex formation, and may have biological consequences beyond cGAS enzymatic activity *per se*. In contrast, a pharmacological approach of cGAS inhibition allows to bypass the potential developmental limitations of genetic deletion models. Specifically, pharmacological inhibition enabled a targeted and temporal control of cGAS activity, thus reducing potential confounding effects on immune maturation and allowing us to study cGAS role in airway inflammation in a more precise manner. Here, we blocked cGAS function with the specific inhibitor RU.521,^20^ which protected mice from BPE allergen-induced airway inflammation and type 2 immune responses. cGAS inhibitor RU.521 treatments had no effect on the expression of *Tmem173* and *Mb21d1* genes (Figure S2), indicating that STING and cGAS overexpression after BPE challenge are not mediated by cGAS activation itself. This is in line with the fact that type I IFNβ was only partially reduced in BPE challenged STING- or cGAS-deficient mice, and speaks against an amplification loop mediated by sGAS/STING induction of type I IFN and ISG expression, among which cGAS *Mb21d1* gene. The field of STING antagonists or cGAS inhibitors has been exploding recently, and the translation to clinical applications is in progress in several areas.^21^^,^^22^^,^^23^ The pharmacological approach contributed novel insights into the therapeutic potential of cGAS inhibition and helps to clarify the importance of the pathway in the mature immune system inflammatory response.

Thus, self-DNA release in the airways is a hallmark of experimental allergen challenge, irrespective of adjuvant usage. DNA sensing by cGAS functionally contributes to the type 2 immune responses and airway hyperreponsiveness and may represent potential therapeutic targets for birch pollen induced allergic lung inflammation.

### Limitations of the study

While our study demonstrates that BPE-induced type 2 response depends on a functional cGAS/STING pathway, we cannot rule out the potential contribution of other DNA sensors, such as DAI/ZBP1 and AIM2. The use of the cGAS inhibitor RU.521 showed promising results to protect against BPE-induced inflammation and mucus production. However, it did not fully prevent inflammation, suggesting the involvement of additional pathways. Furthermore, the relative contributions of cGAS and STING pathways warrants further investigation in future studies, especially in the clinical context of human allergic asthma. Further studies are also needed to delineate the role of cGAS- and STING-independent signaling in allergic airway inflammation.

## Resource availability

### Lead contact

Further information and requests for resources and reagents should be directed to and will be fulfilled by the lead contact, Valerie F.J. Quesniaux (quesniaux@cnrs-orleans.fr).

### Materials availability

All requests for materials used in this article should be directed to and will be fulfilled by the lead contact, Valerie F.J. Quesniaux (quesniaux@cnrs-orleans.fr).

### Data and code availability

• The data reported are available from lead contact on reasonable request.
• This paper does not report original code.
• Any additional information required to reanalyze the data reported in this work paper is available from the lead contact upon request.

## Acknowledgments

This work was supported by CNRS, 10.13039/501100005696University of Orlans, the 10.13039/501100000780European Union funding in Region Centre-Val de Loire (ERDF/ESF N°2024-00012066 Exposome & Inflammation), Region Centre Val de Loire (APR-IR ExAsPIR17 N° 2021-00144721), 10.13039/501100002915Fondation pour la Recherche Médicale (EQU202003010405), and Conseil Général 45 to Q.M. PhD fellowship.

## Author contributions

P.C., M.M., Y.M-N., E.C., Q.M., L.F., N.R., A.L., and S.R., conducted the experiments and analyzed the results; B.R., L.A., V.F.J.Q., and D.T. designed the experiments, discussed the results and wrote the paper.

## Declaration of interests

The authors declare no competing interests.

## STAR★Methods

### Key resources table

**Table:** 

REAGENT or RESOURCE	SOURCE	IDENTIFIER
**Antibodies**		

rat anti-mouse CD45 (clone 30F11)	BD Biosciences	557235; RRID: AB_396609
hamster anti-mouse TCRβ (clone H57-597)	eBiosciences	48-5961-80; RRID: AB_11062012
anti-CD4 (clone RM4-5)	BD Biosciences	552775; RRID: AB_394461
rat anti-mouse CD8α (clone 53-6.7)	BD Biosciences	561967; RRID: AB_10893346
rat anti-mouse IL-4 (clone 11B11)	BD Biosciences	554436; RRID: AB_398556
rat anti-mouse IL-13 (clone ebio13A)	eBiosciences	50-7133-82; RRID: AB_2574279
anti-FcεR1 (clone MAR-1)	eBiosciences	12-5898(-82); RRID: AB_466028
goat anti-rabbit-IgG-HRP-conjugate	Cell signaling	7074; RRID: AB_2099233
horse anti-mouse-IgG-HRP-conjugate	Cell signaling	7076; RRID: AB_330924

**Biological samples**		

Lung	Homemade tissue	
Bronchoalveolar lavage fluid	Homemade tissue	

**Chemicals, peptides, and recombinant proteins**		

RU.521	Invivogen	inh-ru521
Cytofix/cytoperm kit	BD Biosciences	554714; RRID: AB_2869008
DNase I	Sigma	DN25-1G
Liberase	Sigma	5 401 127 001
Trizol	Sigma	15596026
PMA (phorbol 12-myristate 13-acetate)	Sigma-Aldrich	P1585
Ionomycin	Sigma-Aldrich	I9657
Brefeldin A	Ozyme	BLE420601

**Critical commercial assays**		

GoScript™ Reverse Transcription System	Promega	A5001
GoTaq® qPCR Master Mix	Promega	A6002
Quant-iT™ PicoGreen™ dsDNA	ThermoFisher	P11496
IFN alpha/IFN beta Panel ProcartaPlex Mouse	Life Technologies	EPX020-22187-901
Mouse CCL11 DuoSet	R&D systems	DY420
Mouse CCL24 Duoset	R&D systems	DY528
Mouse Amphiregulin (AREG) Duoset	R&D systems	DY989
IL-4, IL-5, IL-13 and IFNγ	Merck Millipore	MCYTOMAG-70K-04
IL-33 Mouse ProcartaPlex™ Simplex Kit	ThermoFisher	EPX010-26025-901
Birch pollen extract (Betula pendula	Stallergenes Greer	XP527D3A25
House dust mite extract	Stallergenes Greer	XPB70D3A25
Ovalbumin	Sigma	A5503-5G

**Experimental models: Organisms/strains**		

Mouse C57BL/6 STING^−/−^	Ahn et al.^2^	
Mouse C57BL/6 cGAS^−/−^	Li et al.^24^	

**Oligonucleotides**		

Mm_Mb21d1_1_SG QuantiTect Primer Assay	Qiagen	QT00131929
Mm_Tmem173_1_SG QuantiTect Primer Assay	Qiagen	QT00261590
Mm_Rn18s_3_SG QuantiTect Primer Assay	Qiagen	QT02448075
Mm_Hprt_1_SG QuantiTect Primer Assay	Qiagen	QT00166768
*Muc5ac (forward: 5′CAGCCGAGA* *GGAGGGTTTGATCT and reverse: 5′AGTCTCTCTCCGCTCCTCTCA*)	Sigma	
*Gob5 forward: (5′-CTGTCTTCCTCTTGAT* *CCTCCA-3′ and reverse: 5′-CGTGGTC* *TATGGCGATGACG-3′)*	Sigma	
Mm_ *hprt1_1_SG_ QuantiTect Primer Assay*	Qiagen	QT00166768

**Software and algorithms**		

Graphpad Prism version 10	Graphpad Software, San Diego, USA	https://www.graphpad.com/; SCR_002798
Bio-Plex Manager software	Bio-Rad	Bio-rad, Bio-Plex Manager; SCR_014330
FlowJo software	TreeStar, Mountain View, CA	https://www.flowjo.com; SCR_008520
ImageJ software	U. S. National Institutes of Health	https://imagej.net/software/imagej/; SCR_003070

### Experimental model and study participant details

#### Mice

Wild-type (WT) C57BL/6 mice and mice deficient for STING (STING^−/−^)^2^ or cGAS (cGAS^−/−^),^24^ were bred in our specific pathogen free animal facility at CNRS (TAAM UAR44, Orleans, France). They were maintained in a 12h light-dark cycle with food and water *ad libitum*, following European and local legislation. For experiments, adult (8–12 week old) animals were kept in ventilated cages and monitored daily. All animal experiments complied with the French Government animal experiment regulations and ARRIVE guidelines. The protocols were submitted to the “Ethics Committee for Animal Experimentation of CNRS Campus Orleans” (CCO) under numbers CLE CCO 2015-1085 and CLE CCO 2019-2017, and approved by the French Minister under APAFIS #19228 and #25876.

### Method details

#### Mouse model of allergic airway disease

For BPE models, mice were immunized by intraperitoneal route with BPE (10 μg/mouse, Greer Laboratories, Lenoir, USA) on day 0 and 14, and challenged with BPE (10 μg in phosphate-buffered saline) daily from day 21–24 by intranasal (i.n.) route under anesthesia. Control mice received saline solution (i.n.).

To inhibit cGAS *in vivo*, a dose response experiment was performed with 0.1, 0.3 and 1 μg/mouse of the cGAS-selective inhibitor RU.521(Vincent et al. ^12^) during sensitization (Day 0 and 14) and challenge (Days 21–24) by i.n. route, 1 h before BPE administration.

Alternatively, for HDM model, mice were sensitized intranasally with 25 μg of house dust mite extract (HDM; Greer Laboratories) under anesthesia on day 0 and 7, followed by three challenges of 10 μg of HDM on days 14–16.

As allergic airway disease involving alum adjuvant, mice were immunized by intraperitoneal route on day 0, 7 and 14 with 20 μg grade V ovalbumin (Sigma) emulsified in 2 mg Alum gel in a total volume of 200 μL and challenged on day 21–24 with 10 μg ovalbumin i.n. as described.^25^ Control mice received saline.

#### Airway hyperresponsiveness

Resistance was recorded with an invasive plethysmograph (Buxco, London, United Kingdom). Mice were anesthetized with intraperitoneal injection of a solution containing ketamine (100 mg/kg; Merial, Lyon, France) and xylazine (10 mg/kg; Bayer, Leverkusen, Germany), paralyzed with D-tubocuranine (0.125%; Sigma), and intubated with an 18-gauge catheter. Respiratory frequency was set at 140 breaths/min with a tidal volume of 0.25 mL. Increasing concentrations of aerosolized methacholine (0, 18.75, 37.5, 75, and 150 mg/mL; Sigma) were administered. Baseline resistance was restored before administering the subsequent doses of methacholine.

#### Bronchoalveolar lavage

Bronchoalveolar lavage (BAL) was performed by washing the lungs four times with 0.5 mL of saline (NaCl 0.9%) solution at room temperature. After BAL collection and centrifugation at 400*g* for 10 min at 4°C, the cell-free BAL fluid (BALF) of the first lavage was stored at −20°C for cytokine analysis. Differential cell counts were performed after May–Grünwald Giemsa (MGG) staining of BAL cellular fractions on cytospins.

#### Measurement of double-stranded DNA

dsDNA was measured in the BALF using Quant-iT PicoGreen dsDNA reagent (Invitrogen, Carlsbad, CA), according to the manufacturer’s protocol.

#### Measurement of cytokines and chemokines

IFNβ, IL-4, IL-5, IL-13 and IL-33 concentrations in BALF or/and lung homogenate were determined by multiplex immunoassay according to the manufacturer’s instructions (eBiosciences or Merck Millipore) using MagPix equipment (BioRad, France). AREG (Amphiregulin), CCL11 (Eotaxin1), and CCL24 (Eotaxin2) concentrations in the BALF or/and lung homogenates were determined by enzyme-linked immunosorbent assay (ELISA) according to the manufacturer’s recommendations (R&D Systems).

#### Quantitative RT-qPCR analysis

Total RNA was collected in RNAlater solution for 24 h at 4°C and snap-frozen in liquid nitrogen. Total RNA was isolated from homogenized mouse lung by using in TRI-Reagent (Sigma) and quantified by using NanoDrop (Nd-1000). Reverse transcription was performed with SuperScriptIII Kit (Invitrogen, Carlsbad, Calif). cDNA was subjected to quantitative real-time PCR using primers for mouse *Tmem173* (forward: 5′TGGCTGCTGATGCCATACTC and reverse: 5′AGACTCGGGGACATCTTCCA; Sigma)*, Mb21d1* (QT00131929, Quiagen)*, Muc5ac* (forward: 5′CAGCCGAGAGGAGGGTTTGATCT and reverse: 5′AGTCTCTCTCCGCTCCTCTCA; Sigma), and *gob5* (forward: 5′CTG TCT TCC TCT TGA TCC TCC A and reverse: 5′CGT GGT CTA TGG CGA TGA CG; Sigma) and GoTaq qPCR-Master Mix (Promega). RNA expression was normalized to *hprt1* (QT00166768, Quiagen) *or RN18S* (QT02448075, Quiagen) expression. Data were analyzed using the comparative analysis of relative expression, by 2^ΔCt^ methods.^26^

#### Lung mononuclear cells isolation

Lung mononuclear cells were isolated from mice 24 h after the last challenge. Briefly, the aorta and inferior vena cava were sectioned, and the lungs were perfused with saline. The lungs were sliced into small cubes and then incubated for 45 min in 2 mL of RPMI 1640 solution and digested in 125 mg/mL Liberase TL and 1 mg/mL DNAse 1 (Roche Diagnostics). Isolated lung mononuclear single cells were plated in round-bottom 96-well plates (2 × 10^6^/mL) and restimulated for 2.5 h *in vitro* with phorbol 12-myristate 13-acetate (50 ng/mL) and ionomycin (750 ng/mL, both from Sigma-Aldrich) in the presence of Brefeldin A (1 μg/mL, BD Biosciences) for intracellular flow cytometric analysis.

#### Flow cytometry

Lung mononuclear cells were stained with the following antibodies: rat anti-mouse CD45 (clone 30F11), hamster anti-mouse TCRβ (clone H57-597), rat anti-mouse CD4 (clone RM4-5), rat anti-mouse CD8α (clone 53-6.7), rat anti-mouse IL-4 (clone 11B11), rat anti-mouse IL-13 (clone ebio13A). For extracellular staining, single cells (2.10^6^ cells/well) were incubated with 2.4G2 Fc-receptor antibodies to reduce nonspecific binding and stained at 4°C with antibodies for 20 min. Intracellular staining was performed following extracellular staining and permeabilization for 20 min with cytofix/cytoperm kit (BD Biosciences).

Data were acquired using FACS Canto II flow cytometer. Fluorescence data were acquired by using DIVA software (BD Bioscience, France) and analyzed using FlowJO software (Treestar).

#### Lung histology

Lungs were fixed in 4% buffered formaldehyde and 3 μm sections were stained with periodic acid Schiff reagent (PAS) and examined with a Leica DM6000B microscope (×20 magnifications, scale bar 100 μm). Peribronchial infiltrates and mucus hypersecretion were assessed by a semi-quantitative score (0–5) by two observers independently.

### Quantification and statistical analysis

#### Statistical analysis

Statistical analyses were performed with GraphPad Prism Version 10 (GraphPad Software Inc., San Diego California USA). All statistical analyses were preceded by Shapiro-Wilk or Agostino & Pearson normality tests, followed by the recommended test according to the results of Normality test. Statistical significance was determined by one-way ANOVA followed by a Sidak post-test, Kruskal Wallis followed by Dunns multiple comparison post-test or two-way ANOVA followed by Dunnett’s multiple comparison test as indicated in the figure legends. Outliers were detected using the Grubb’s method. Spearman test was used for correlation analysis. Data are expressed as mean ± SEM. Statistically significant differences were defined as *p* value < 0.05 (∗), *p* value < 0.01 (∗∗), *p* value < 0.001 (∗∗∗) and *p* value < 0.0001 (∗∗∗∗).
