# Supplementary material for: Birch pollen allergen-induced dsDNA release activates cGAS-STING signaling and type 2 immune response in mice
Source: iScience. 2025 Mar 31;28(5):112324. doi: 10.1016/j.isci.2025.112324 (PMC12018559; doi:10.1016/j.isci.2025.112324)
Supplement: Document S1. Figures S1 and S2 [file mmc1.pdf]

iScience, Volume *28*

Supplemental information

Birch pollen allergen-induced dsDNA release

activates cGAS-STING signaling

and type 2 immune response in mice

Pauline Chenuet, Manon Mellier, Yasmine Messaoud-Nacer, Elodie Culerier, Quentin Marquant, Louis Fauconnier, Nathalie Rouxel, Aurélie Ledru, Stéphanie Rose, Bernhard Ryffel, Lionel Apetoh, Valérie F.J. Quesniaux, and Dieudonnée Togbe

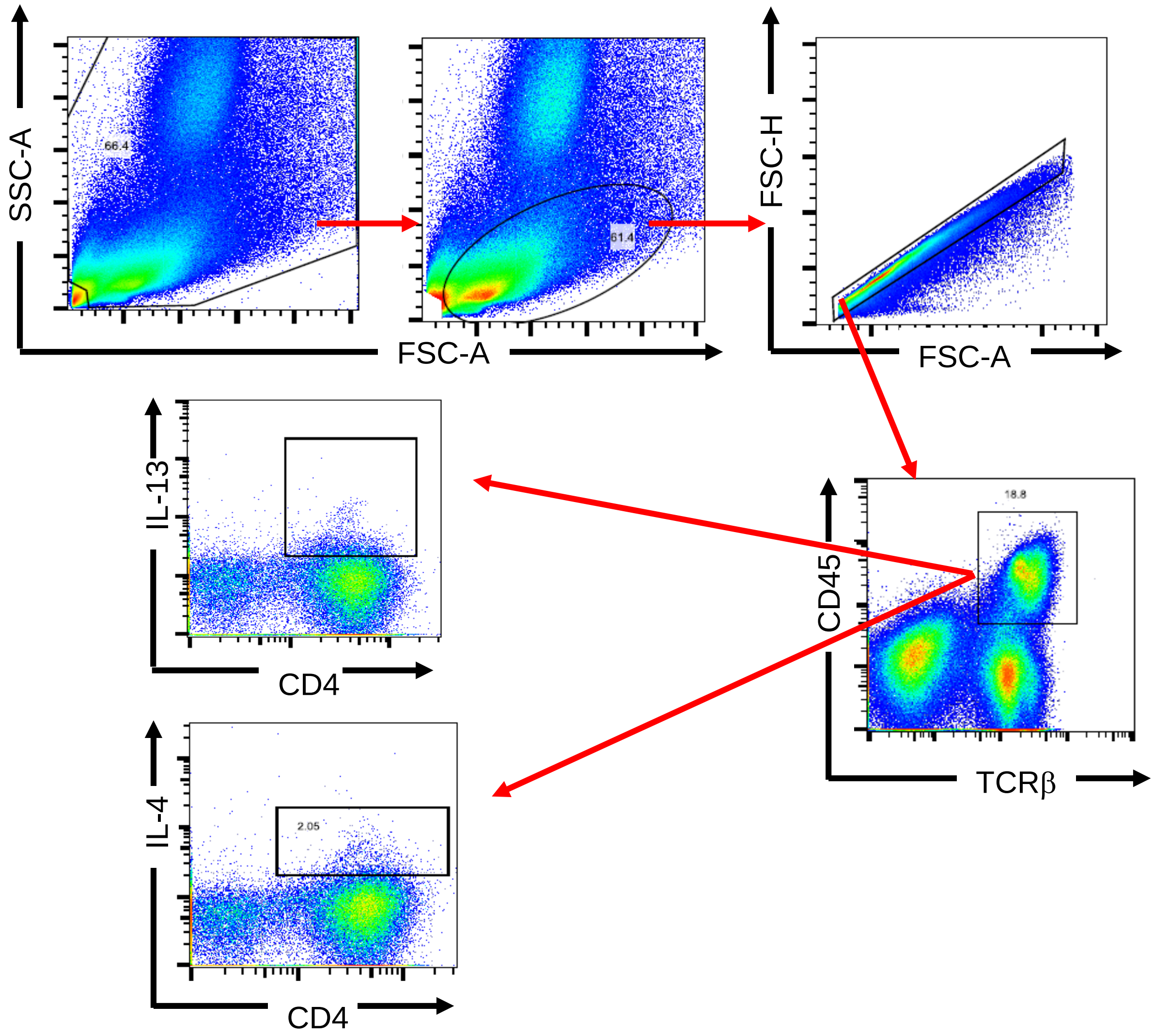

Figures S1:

Gating strategy for the flow cytometry analysis of IL-13 and IL-4 producing CD4+ Th2 cells. Related to Figure 2 K-N

Representative dot plots of control WT mice receiving birch pollen.

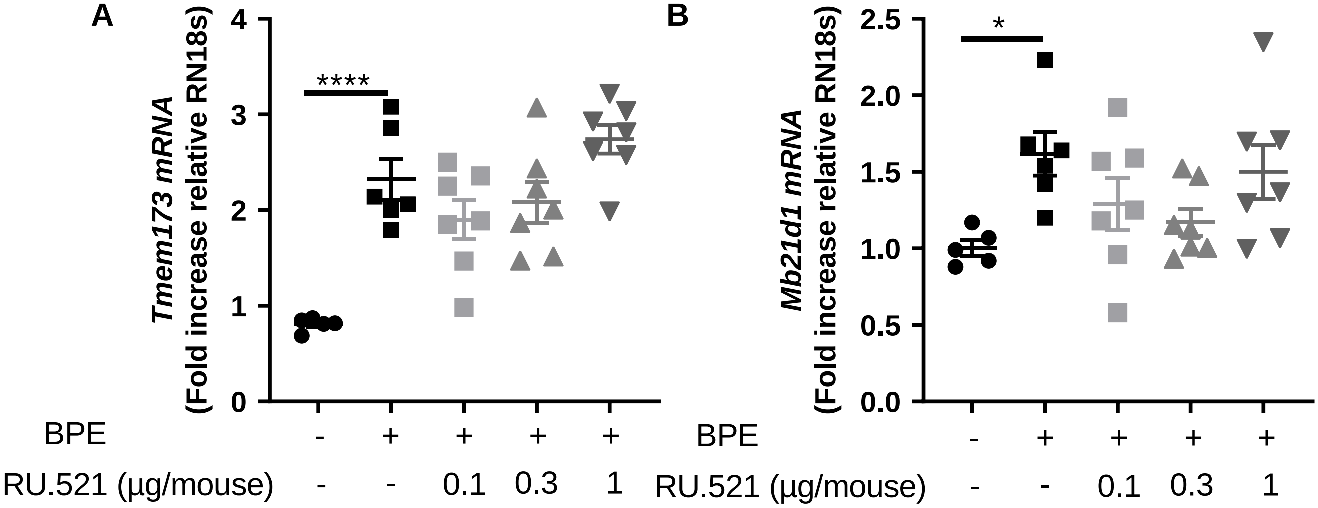

Figure S2: Pulmonary expression of STING and cGAS transcripts under cGAS inhibition by RU.521. Related to Figure 4.

Expression of STING *Tmem173* and cGAS *Mb21d1* transcripts in the lungs normalized to *RN18s* expression.
